# Echoes of Childhood Trauma: A Rare Case of Holmes Tremor With Neuroanatomic Imaging and Video Evidence

**DOI:** 10.7759/cureus.98408

**Published:** 2025-12-03

**Authors:** Ridhika Prasad, Dalia Gazallo, Robert J Coni

**Affiliations:** 1 School of Medicine, Burrell College of Osteopathic Medicine, Las Cruces, USA; 2 Preclinical Medicine, Burrell College of Osteopathic Medicine, Melbourne, USA

**Keywords:** clinical neuroanatomy, closed head injury, holmes tremor, levodopa-carbidopa, neurology case report, post-traumatic movement disorder

## Abstract

Neurologic tremors constitute a heterogeneous group of movement disorders that are often challenging to classify due to overlapping clinical presentations. While Parkinsonian, essential, and cerebellar tremors are among the most frequently encountered types, rarer causes such as thalamic infarction may be easily overlooked. We describe a rare case of tremor secondary to thalamic infarction, emphasizing its relevance as an uncommon yet clinically important etiology within the spectrum of neurologic tremors. Focal midbrain lesions can produce a distinctive coarse tremor, referred to as Holmes or rubral tremor, arising from disruption of the dentatorubrothalamic pathway. We report the case of a 53-year-old right-handed woman who presented with a left-sided, 4-Hz coarse tremor evident at rest and with action, demonstrating partial responsiveness to levodopa therapy. The tremor began shortly after a closed head injury at age six, accompanied by left-sided numbness and persistent motor control difficulties beginning within a few weeks. An MRI in 1997 revealed a small infarct-like lesion in the right thalamus, unchanged on a 2022 MRI. Misdiagnosed initially as a conversion reaction, she later received a diagnosis of "essential tremor with Parkinsonian features" and was treated with primidone and intermittent carbidopa-levodopa. The tremor impacts her work as a dental hygienist, but levodopa provides partial relief. Holmes tremor, characterized by low-frequency (2-4 Hz) high-amplitude tremors at rest, with movement, or in posture, is distinct from essential and cerebellar tremors. This case illustrates the rarity of this particular tremor, highlights its distinction from other tremors, and demonstrates potential treatments, including levodopa, thalamotomy, and deep brain stimulation.

## Introduction

Holmes tremor (HT), also called “rubral tremor,” was first described in 1904 by Dr. Gordon Morgan Holmes and is a rare and complex clinical phenomenon characterized by an irregular, low-frequency tremor (2-4 Hz) with high amplitude. This tremor may manifest as resting, postural, or intentional components, either independently or concurrently, and it predominantly involves the proximal limbs [1]. Unlike essential tremor, which is higher in frequency (6-12 Hz) and affects multiple body parts, or cerebellar tremor, which is similarly low-frequency (<5 Hz) but primarily occurs during voluntary movements or while maintaining posture, HT presents a unique diagnostic challenge [2].

The etiology of HT is diverse, encompassing traumatic injuries, infections, demyelination, and ischemic or hemorrhagic cerebrovascular disorders [1]. It typically emerges following a latent period of 1 to 24 months after an initial neurological insult, during which time the tremor begins to develop. HT is frequently attributed to structural lesions within the upper brainstem, thalamus, or cerebellum, disrupting the cerebellar outflow pathways [3]. Although historically referred to as “rubral tremor,” its pathogenesis extends beyond isolated damage to the red nucleus, as lesions in the thalamus and upper brainstem also contribute to its development. Notably, thalamic lesions can induce high-amplitude, low-frequency tremors, underscoring the thalamus's critical role in the "long-loop" pathway. Additionally, the involvement of the nigrostriatal pathway is speculative and supported by observations of the tremor’s responsiveness to levodopa.

HT is exceedingly rare, with only approximately 160 cases reported in the literature [1], and much of the current understanding is derived from case reports and small case series. Despite being recognized for over a century, HT is often misdiagnosed due to its heterogeneous presentation and overlap with other tremor disorders [4]. This diagnostic complexity, combined with the multifaceted nature of its underlying mechanisms, renders its treatment particularly challenging in clinical practice.

## Case presentation

**Figure 1:**
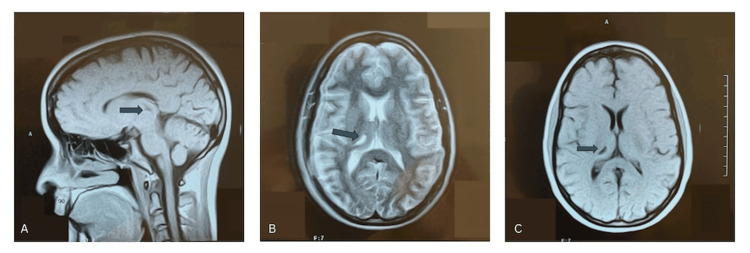
1997 MRI Brain Imaging (A) Coronal T1-weighted sequence; (B) Axial T2-weighted sequence; (C) Axial T1-weighted sequence.

**Figure 2:**
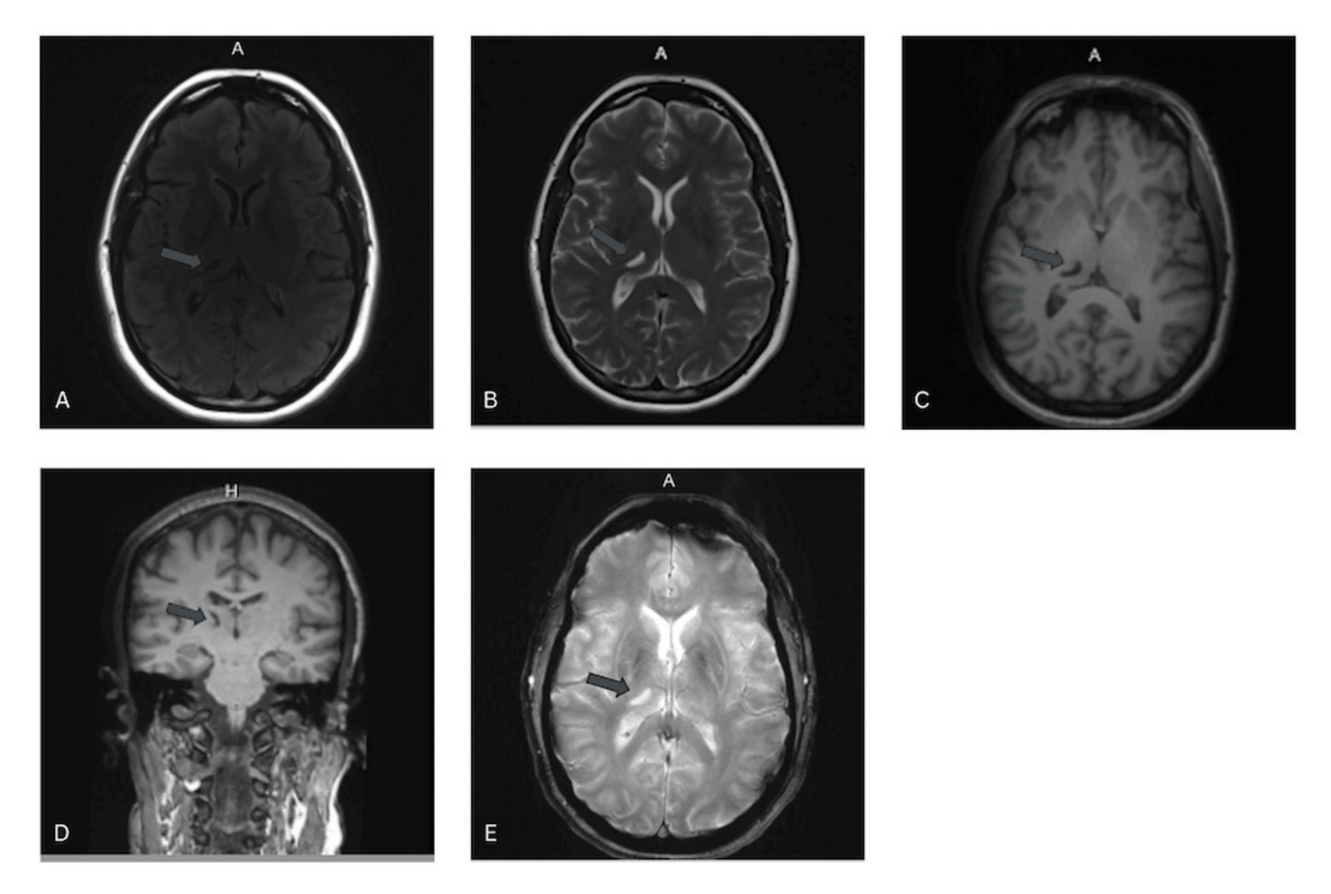
2022 MRI Brain Imaging (A) Axial T2 fluid-attenuated inversion recovery (FLAIR)-weighted magnetic resonance image; (B) Axial T2-weighted sequence; (C) Axial T1-weighted sequence; (D) Sagittal T1-weighted sequence; (E) Axial susceptibility heme-weighted sequence.

A 53-year-old right-handed woman presented to our clinic to establish local neurological care after relocating. She had previously been diagnosed with “essential tremor with Parkinsonian features” and was taking primidone 50 mg daily along with intermittent doses of carbidopa-levodopa. Her tremor primarily affected the left side of her body and had been present since she was six years old. The onset of her symptoms followed a closed head injury that occurred when she was struck on the head by a ball while playing, causing her to fall and lose consciousness. She recalled experiencing immediate left-sided numbness and difficulty with motor control, which gradually evolved into a persistent tremor. A few weeks after the injury, a physician attributed her left-sided weakness to a probable conversion reaction, though medical records from that time are unavailable. At the age of 26, after becoming financially independent, she underwent a brain magnetic resonance imaging (MRI) that revealed a small infarct-like lesion within the right thalamus on both T1- and T2-weighted images (Figures 1A-1C). A repeat MRI performed in 2022 demonstrated that these thalamic lesions remained stable with no new abnormalities identified (Figures 2A-2E).

The patient is employed as a dental hygienist and currently manages her tremor with a regimen of carbidopa-levodopa (25/100 mg) administered 5-6 times daily during work hours. She reports that stabilizing her left arm against her torso while leaning over patients provides partial functional compensation and reduces the amplitude of her tremor. A comprehensive workup for Wilson’s disease, including serum ceruloplasmin and copper levels, was within normal limits, effectively excluding this etiology. An accompanying video (Video 1) demonstrates the classical features of the tremor as described.

**Video 1:** Manifestation of Holmes tremor in our patient.

At the age of 40, she was diagnosed with Lyme disease, which she associates with a subsequent exacerbation of tremor severity. Her family history is notable for cerebrovascular disease, as her sister suffered a stroke at the age of two. The patient believes this familial history contributed to her earlier diagnosis of a conversion reaction. However, repeat neuroimaging revealed no radiographic evidence of prior cerebrovascular insult, vascular malformation, or hemosiderin deposition, supporting the conclusion that stroke is unlikely to have played a role in the pathogenesis of her tremor. Instead, the close temporal association between the head trauma and symptom onset strongly suggests a post-traumatic etiology. 

## Discussion

A 53-year-old right-handed woman presented with a distinctive left-sided, 4-Hz coarse tremor exhibiting both rest and action components, accompanied by partial but meaningful symptomatic responsiveness to levodopa therapy. The immediate onset of tremor in this patient following childhood head trauma is most consistent with a selective traumatic injury to the dentatorubrothalamic and adjacent dopaminergic midbrain pathways, which can produce tremor as the earliest and predominant neurological manifestation. Although HT is classically described as having a delayed onset after structural brain injury, selective tract disruption from trauma may present abruptly without accompanying cortical or sensory deficits. In this case, heme-susceptibility MRI shows no evidence of prior hemorrhage, infarction, or vascular malformation, making a stroke-related etiology unlikely, particularly since ischemic HT typically appears months after the vascular event and is frequently associated with additional focal neurologic findings. The patient’s history, being struck in the head with immediate loss of consciousness and awakening with a coarse, low-frequency tremor, and the lifelong stability of her symptoms over 45 years further support a static post-traumatic cerebellothalamic injury, rather than an ischemic process, as the underlying mechanism. The dentatorubrothalamic pathway, crucial in coordinating motor control, begins in the cerebellar dentate nucleus. Fibers travel through the superior cerebellar peduncle, decussating in the midbrain near the red nucleus, before projecting to the ventrolateral (VL) nucleus of the thalamus. The VL thalamus communicates with the motor cortex to facilitate smooth movements. HT often arises after damage to the midbrain, thalamus, or the pathway itself, particularly involving the red nucleus or VL nucleus, as seen in our patient’s lesion [4-6].

HT may be affected indirectly by a lesion in the thalamus. The centromedian (CM) nucleus, a key structure within the thalamus, serves as a critical hub for relaying and integrating neural signals. It projects to important subcortical structures such as the caudate nucleus, putamen, and globus pallidus, which are integral to motor control and cognitive processing. Functionally, the CM nucleus is deeply involved in motor regulation, where it contributes to the fine-tuning of movement by facilitating communication within the basal ganglia-thalamocortical circuitry [5,6]. Beyond motor control, it plays a role in cognitive functions, such as attention, decision-making, and behavioral adaptation, highlighting its diverse responsibilities in neural regulation. In the context of HT, the CM nucleus may indirectly influence the condition through its connections with the basal ganglia and its role in integrating cerebellar and motor cortical inputs. Disruption of thalamic circuits, including those involving the CM nucleus, can lead to the dysregulated motor outputs characteristic of tremor disorders, further underscoring its importance in maintaining motor harmony and precision.

While the pulvinar nucleus does not directly contribute to the pathogenesis of HT, its anatomical location and extensive connections suggest a potential role in modulating symptoms when lesions involve neighboring structures. The pulvinar is situated adjacent to the VL nucleus of the thalamus, where the dentatorubrothalamic tract (DRT) terminates, making it vulnerable to compression or extension of lesions in this region. Functionally, the pulvinar links to other thalamic nuclei and the cerebral cortex, allowing it to integrate sensory, motor, and cognitive signals. These attributes suggest that damage to the pulvinar, even indirectly, could exacerbate motor and sensory dysfunctions, potentially influencing the overall clinical presentation of HT [7,8].

The patient was treated with levodopa therapy as a result of her tremor. While carbidopa-levodopa does not directly target the DRT, it exerts its effects by modulating the thalamic components of this pathway, where motor information from both the cerebellum and basal ganglia converges. The basal ganglia, particularly through the indirect and direct pathways, play a critical role in motor control, influencing the fine-tuning of movements and motor output. Carbidopa-levodopa primarily affects dopaminergic signaling in the basal ganglia, improving motor coordination by enhancing the output of the motor cortex and modulating the interaction between the basal ganglia, cerebellum, and thalamus [5-7].

A double lesion theory has been proposed in the literature, suggesting that damage to both the dopaminergic nigrostriatal system and the cerebellothalamocortical (or dentatorubroolivary) pathways is necessary to produce HT, and may explain the tremor’s partial responsiveness to dopaminergic therapies such as levodopa [5]. This theory, supported by cases where combined lesions are observed, posits that disruption of the dopaminergic system contributes to resting tremor components, while cerebellothalamic damage accounts for postural and kinetic tremor elements. Patients have been documented with dual pathway injuries demonstrating classic HT features and better response to dopamine replacement [6].

However, not all cases align with this dual-lesion hypothesis. Several reports argue that a single lesion, typically affecting the thalamus, midbrain, or cerebellar outflow tract, is sufficient to produce HT, particularly if the lesion affects integrative nodes like the ventrolateral thalamic nucleus [7]. Our patient, who demonstrates a stable, isolated right thalamic lesion with no evidence of basal ganglia or nigrostriatal system involvement, provides compelling support for this perspective.

The pathophysiological mechanism may involve diaschisis, in which a lesion in the thalamus indirectly disrupts activity in distant but functionally connected regions like the red nucleus or basal ganglia. This can result in motor circuit dysregulation and generation of a tremor that mimics a multifocal origin, despite the presence of a single identifiable lesion. Such functional disconnections may explain the heterogeneity in symptom onset, frequency, and response to therapy observed in HT cases.

## Conclusions

We present a case of Holmes (rubral) tremor, a rare presentation of a movement disorder characterized by a combination of rest, postural, and action tremor resulting from disruption of the dentatorubrothalamic pathway. MRI scans revealed a lesion localized to the ventrolateral nucleus of the right thalamus, consistent with known anatomical correlates. The patient’s history included childhood head trauma followed by persistent left-sided weakness and numbness, with imaging and laboratory studies effectively excluding alternative etiologies such as tumor, vascular malformations, demyelinating disease, or metabolic and genetic causes. Given the temporal relationship between trauma and symptom onset, a post-traumatic origin is the most likely explanation. The accompanying video demonstrates a unilateral left-sided tremor, corresponding to the contralateral right thalamic lesion, with features evident at rest, accentuated with posture, and further exaggerated with intentional movement at a frequency of approximately 3.7 Hz, findings that are characteristic of Holmes tremor and help distinguish it from other movement disorders.

This case underscores the diagnostic and therapeutic challenges of HT. The patient has experienced partial symptomatic relief with levodopa therapy, consistent with previous observations, while other modalities such as stereotactic thalamotomy and deep brain stimulation have shown variable success in similar cases. Functionally, her role as a dental hygienist has been complicated by compensatory postures used to stabilize her tremor, leading to significant cervical spondylotic changes, chronic discomfort, and reduced endurance. Earlier recognition of the disorder could have allowed for timely consideration of surgical options and ergonomic interventions to improve her quality of life.
